# Reexamining the frequency range of hearing in silver (*Hypophthalmichthys molitrix*) and bighead (*H*. *nobilis*) carp

**DOI:** 10.1371/journal.pone.0192561

**Published:** 2018-03-09

**Authors:** Brooke J. Vetter, Marybeth K. Brey, Allen F. Mensinger

**Affiliations:** 1 Biology Department, University of Minnesota Duluth, Duluth, MN, United States of America; 2 Department of Psychology, University of Washington, Seattle, WA, United States of America; 3 U.S. Geological Survey, Upper Midwest Environmental Sciences Center, La Crosse, Wisconsin, United States of America; National Institutes of Health, UNITED STATES

## Abstract

Silver (*Hypophthalmichthys molitrix*) and bighead (*H*. *nobilis*) carp (collectively bigheaded carp) are invasive fish that threaten aquatic ecosystems in the upper Midwest United States and the Laurentian Great Lakes. Controlling bigheaded carp is a priority of fisheries managers and one area of focus involves developing acoustic deterrents to prevent upstream migration. For an acoustic deterrent to be effective however, the hearing ability of bigheaded carp must be characterized. A previous study showed that bigheaded carp detected sound up to 3 kHz but this range is narrower than what has been reported for other ostariophysans. Therefore, silver and bighead carp frequency detection was evaluated in response to 100 Hz to 9 kHz using auditory evoked potentials (AEPs). AEPs were recorded from 100 Hz to 5 kHz. The lowest thresholds were at 500 Hz for both species (silver carp threshold: 80.6 ± 3.29 dB re 1 μPa SPL_rms_, bighead carp threshold: 90.5 ± 5.75 dB re 1 μPa SPL_rms_; mean ± SD). These results provide fisheries managers with better insight on effective acoustic stimuli for deterrent systems, however, to fully determine bigheaded carp hearing abilities, these results need to be compared with behavioral assessments.

## Introduction

Silver (*Hypophthalmichthys molitrix*) and bighead (*H*. *nobilis*) carp (collectively bigheaded carp) are invasive to the Mississippi River Drainage and these prodigious filter feeders threaten native species [1–4] by altering trophic structures in areas where their populations are high [5]. Although their initial introduction in North America was in the southern reaches of the Mississippi River Drainage, bigheaded carp have since migrated north and now threaten the Laurentian Great Lakes via the Chicago Sanitary and Ship Canal [6–8]. There is currently an electric dispersal barrier in the Chicago Sanitary and Ship Canal that separates Lake Michigan from the Illinois and Des Plaines Rivers, however, this barrier is costly and must be operated continuously to prevent further northward migration [9]. Therefore, alternate non-physical deterrents have been proposed both as a backup during maintenance of the electric barrier and to be implemented in additional areas of concern, such as lock chambers.

One promising non-physical barrier is an acoustic deterrent, either used alone [10] or in combination with bubbles and/or strobe lights [11, 12]. Bigheaded carp are ostariophysans and possess Weberian ossicles, which are bony structures that transmit vibrations from the swim bladder to the inner ear and allow sensitivity to higher frequencies [13]. Bigheaded carp displayed negative phonotaxic behavior in response to an outboard motor recording (0.06–10 kHz), suggesting both species can be deterred by broadband sound[14, 15]; however which portion of the frequency spectrum the fish were reacting to is unclear; thus further assessment of bigheaded carp hearing was warranted. Lovell et al. [16] examined auditory evoked potentials (AEPs) for silver and bighead carp and reported AEPs could be stimulated by 3 kHz tones but did not examine higher frequencies. As AEPs have been recorded above 3 kHz in other ostariophysans (see [17] for a review on AEP studies), the purpose of this study was to determine if silver and bighead carp have greater frequency range than previously reported to aid in the optimization of acoustic deterrents.

The AEP technique was first developed for mammals [18, 19] and adapted for fish by Kenyon et al. [20]. This method uses minimally invasive subcutaneous or cutaneous electrodes to record evoked potentials in response to acoustic stimuli. As electrodes are often placed above the brainstem, there has been some confusion as to the origin of the recorded AEPs, however, it is now believed that in most fish AEP studies, AEPs result from microphonic potentials from hair cells and/or their afferent nerves rather than brainstem activity [21]. As the hair cells have opposite orientation, they produce a characteristic double-frequency response [22] and this is evident in AEPs recorded from fish [23, 24]. It is important to note that while AEP studies provide valuable information about the frequencies that stimulate auditory end organs, they are not a comprehensive assessment of the fish’s hearing ability and can only provide relative thresholds. To determine true frequency sensitivity, behavioral experiments, which assess higher order acoustical processing, must also be conducted [21].

In this study, the range of frequencies that silver and bighead carp can detect was evaluated using the AEP technique. Common carp (*Cyprinus carpio*) were also tested to serve as a reference, since multiple AEP studies have been published on this species [25, 23]. Additionally, although bigheaded carp are ostariophysans and are capable of detecting sound pressure, this study determined threshold curves for both sound pressure and acoustic particle motion, as recommended by Popper and Fay [26]. This information provides a basis on which behavioral assessments can be designed to better understand bigheaded carp hearing and evaluate effective acoustic deterrents.

## Methods

### Animal husbandry

All experiments were conducted at the University of Minnesota Duluth in Duluth, MN. Silver (n = 5; standard length (SL): 13.4 ± 1.2 cm, mean ± 1 SD), bighead (n = 5; 12.3 ± 1.2 cm SL), and common (n = 3; 6.7 ± 0.7 cm SL) carp were obtained in the spring of 2017 from the U.S. Geological Survey (USGS) in Columbia, MO. Silver and bighead carp were maintained in a circular 1230 L (2 m diameter) indoor tank equipped with a biological, chemical, and mechanical filtration system (Fluval FX6 High Performance Canister Filter, Fluval, Baie d’Urfé, Quebéc, Canada) and fed a diet of liquid algae mixture (~300 mL; 1:1 *Chorella* and *Spirulina*; Bulk Foods, Toledo, OH) daily. A Prohibited Invasive Species Permit (#391) from the Minnesota Department of Natural Resources and an Injurious Wildlife Permit (MA-98346B-0) from the U.S. Fish and Wildlife Service were obtained prior to acquisition of the animals and the fish were maintained in a locked room with restricted access. Common carp were housed in an 80 L rectangular tank (1.5 m x 0.25 m x 0.5 m) equipped with the same filtration system and fed goldfish flakes (Tetra Werke; Melle, Germany) daily. Water quality was monitored daily and the temperature ranged between 19 and 22°C for both tanks. All experiments were conducted in accordance with protocol #1604-33658A approved by the Institutional Animal Care and Use Committee of the University of Minnesota.

### Auditory evoked potentials

Prior to electrode implantation, fish were anesthetized using phosphate buffered tricaine methanesulfonate (0.005%; Western Chemical Inc., Ferndale, WA) and a tail pinch was used to ensure that the dosage was effective at anesthetizing the animal. Fish were given an intramuscular injection of the paralytic pancuronium bromide (0.001%; Sigma Aldrich, St Louis, MO) dissolved in 0.9% NaCl (Thermo Fisher Scientific; Waltham, MA) to reduce muscle activity, although opercular movements persisted allowing self-ventilation. Each fish was placed in a mesh sling and suspended in the middle of a 350 L circular tank (88 cm inside diameter, 57 cm water depth) such that the top of the cranium was 4 cm below the water surface and 35 cm above an underwater speaker (UW-30; Lubell Labs Inc.; Whitehall, OH). Water temperature was maintained between 19 and 22°C. Two stainless steel electrodes (Rochester Electro-Medical Inc.; Tampa, FL) were insulated with finger nail polish, except for 1 mm at the tip, and implanted just beneath the surface of the skin between the nostrils (reference electrode) and above the brainstem (recording electrode). Prior to collecting data, electrode placement, which was guided by anatomical markers, such as the location of the eyes and opercular openings, was verified by testing individuals from each species to ensure the magnitude of the AEP at each frequency was consistent. The tank was elevated from the cement floor with cinderblocks (41 x 20 x 10 cm) and a 1 cm thick rubber mat was placed between the tank and cinderblock to dampen vibrations. A four sided frame (110 x 125 x 182 cm) constructed from galvanized angle iron surrounded the tank, with three of the sides and top covered with FOAMULAR Insulation Sheathing (2.54 cm thick; Owens Corning; Toledo, OH) to further reduce noise and to block the fish from seeing the observer.

The AEP signal was amplified with a headstage (gain = 10x; Dagan Corporation; Minneapolis, MN) connected to an extracellular differential amplifier (gain = 100x; Dagan Corporation; Minneapolis, MN) using 20 Hz high pass and 10 kHz low pass filters. The signal was then collected and digitized by a Cambridge Electronic Design data acquisition system (Micro3 1401; CED; Cambridge, UK), which was also used to control the sound presentation. The sound pressure level was controlled with a programmable attenuator (CED 3505; CED; Cambridge, UK). The sound pressure level output from the attenuator was measured and calibrated using a Brüel and Kjaer hydrophone (8103; Brüel and Kjaer; Naerum, Denmark), placed in the same position as the experimental fish. The hydrophone was connected to a Nexus Conditioning Amplifier (2692-01s; Brüel and Kjaer; Naerum, Denmark). Custom Spike2 (version 8; CED; Cambridge, UK) scripts were used to calibrate the attenuator, administer sound stimuli, and collect data during the AEP procedure. The acoustic particle motion at the fish position was measured using a three dimensional accelerometer (sensitivity: 100 mV g^-1^ (10.2mV ms^-2^); model: W356A12/NC, PCB Piezotronics Inc., Depew, NY) modified to be neutrally buoyant and connected to a signal conditioner (482C15, PCB Piezotronics Inc.). The accelerometer was placed such that the x-dimension corresponded to the fish’s anterior/posterior position, the y-dimension was left/right and the z-dimension was dorsal/ventral. Particle motion measurements were obtained in the x, y, and z-axes at all frequencies and sound pressure levels evaluated. These measurements were then individually converted to magnitude vectors. All reported particle motion thresholds were calculated using the following equation: 20log(√(x^2^ + y^2^ + z^2^)), where x, y, and z were the magnitude vectors [27–29]. Finally, as suggested by Popper and Fay [26], the acoustic impedance of the tank, or the ratio of sound pressure level to particle motion level, was determined for three sound pressure levels: 119, 130, and 145 dB re 1 μPa SPL_rms_.

Pure tone bursts (50 ms; 500 repetitions; 3 ms delay) were broadcast to silver and bighead carp between 100 Hz and 9 kHz. The first three silver and bighead carp tested showed inconsistent response to frequencies > 5 kHz and ≤ 7 kHz and no responses to frequencies > 7 kHz to 9 kHz; therefore, subsequent fish were only tested using frequencies from 100 Hz to 5 kHz. The common carp were only tested at 100, 200, 400, 600, 800, 1000, 2000, 4000, and 5000 Hz and served as a reference as there are multiple published tuning curves for this species [25, 23]. Responses were collected and averaged using the Spike2 ABR script (all scripts available at www.ced.co.uk).

The presence of an AEP was verified by two means: (1) through observation of the characteristic wave visible above the background noise, as is commonly used in AEP studies (e.g. [30–32]) and (2) through fast Fourier Transform (FFT) analysis (Hanning window = 1024) to calculate the power spectra of the average waveforms at two times the stimulus frequency, because of the opposed orientation of hair cells [22]. The auditory threshold at each frequency was defined as the lowest sound pressure level that elicited both a repeatable AEP, visible above background noise, and a FFT peak at twice the stimulus frequency. In determining the threshold, AEPs were first elicited using a sound pressure level above threshold (100 Hz to 2 kHz: 130 dB re 1 μPa SPL_rms_; 3 to 5 kHz: 131–147 dB re 1 μPa SPL_rms_). After this initial test, the sound pressure levels were decreased in 3 dB steps for each frequency until an auditory evoked potential could not be determined both visually and via FFT analysis. All fish were sacrificed using an overdose of MS-222 at the end of the study and no AEPs were elicited from a sacrificed fish that served as a “dead control”.

To determine the relative amplitude for the FFT analyses, the raw voltages (μV2) were normalized based on the highest FFT peak. A repeated measures ANOVA with a Holm-Sidak test was used to compare the sound pressure and acceleration thresholds for all three species at each frequency examined using SigmaPlot (version 12.5). All threshold data were normally distributed (Shapiro-Wilk P > 0.05) and are reported as mean ± 1 SD.

## Results

**Fig 1:**
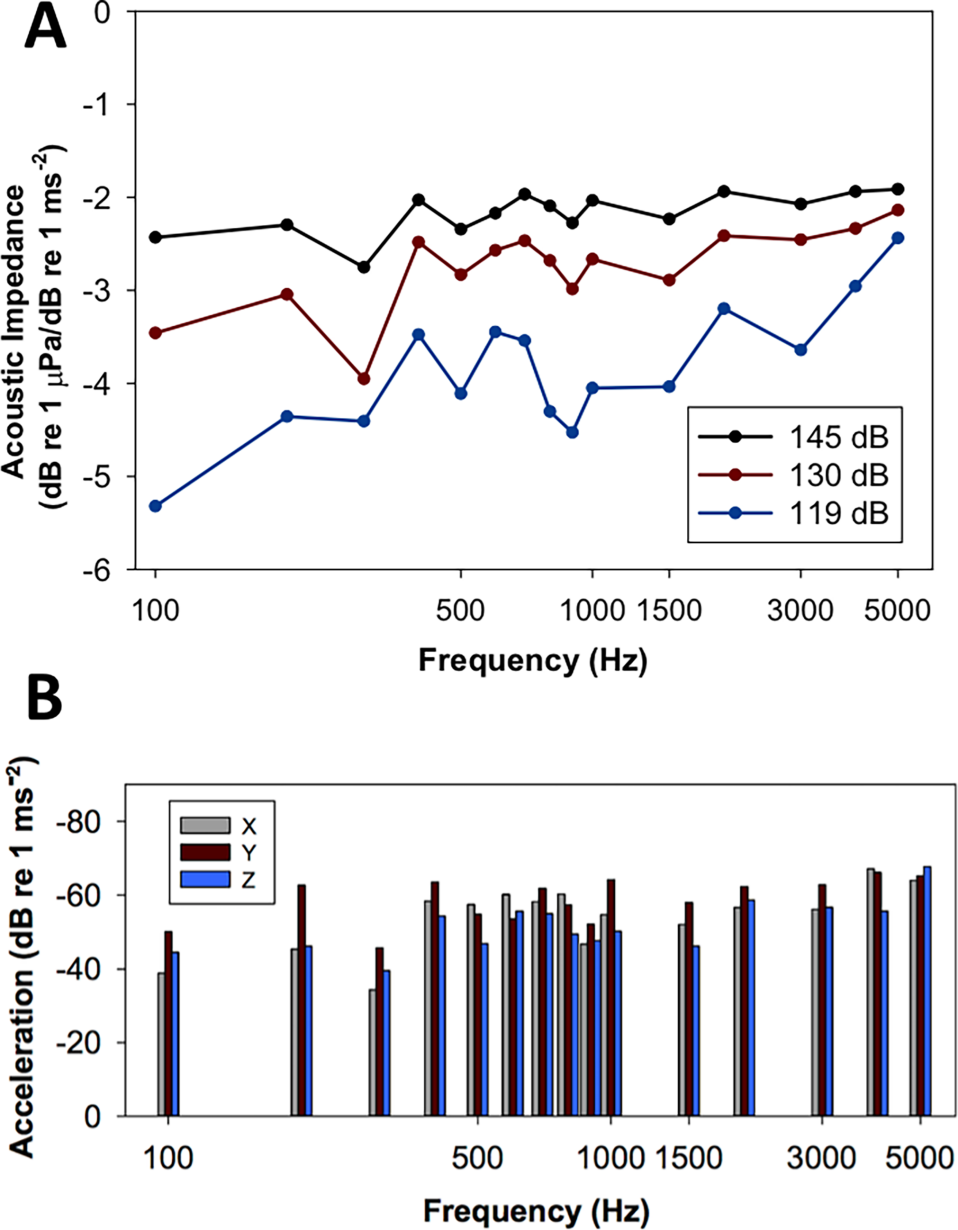
Acoustic characterization of the experimental tank. **A)** Acoustic impedance (ratio of sound pressure level to particle motion level) at three sound pressure levels (119, 130, and 145 dB 1 μPa SPL_rms_) for all frequencies examined. There are no apparent resonances at any of the frequencies. **B)** Particle acceleration levels for each of the x, y, and z magnitude vectors at 130 dB re 1 μPa for all frequencies examined.

**Fig 2:**
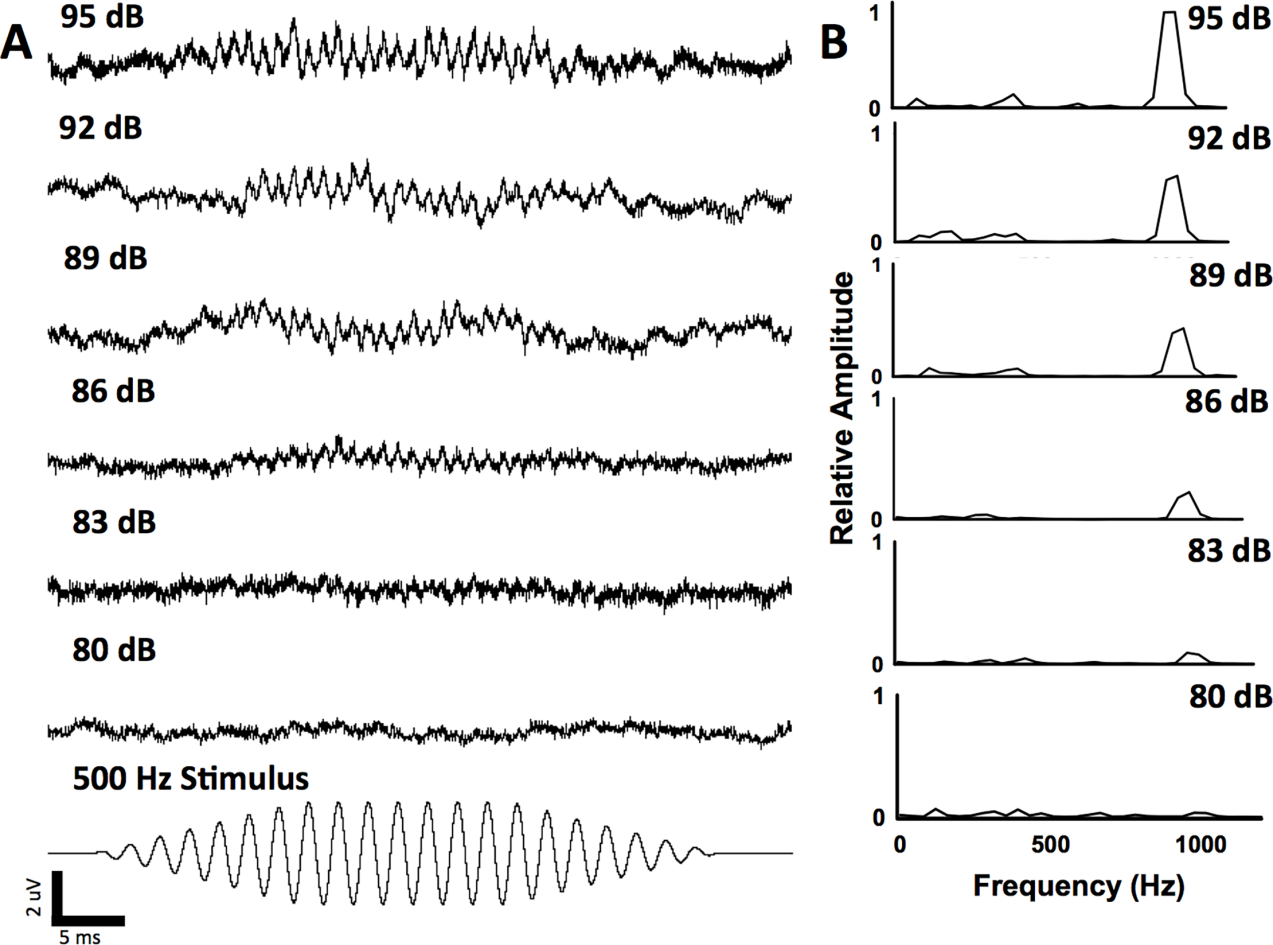
Example auditory evoked potentials (AEPs) recorded from a silver carp at 500 Hz. Averaged AEP traces **(A)** and FFT analysis **(B)** at six different sound pressure levels, including below the hearing threshold (80 dB re 1 μPa SPL_rms_). FFT peaks are two times the stimulus frequency (1000 Hz). Hearing threshold was 83 dB re 1 μPa SPL_rms_ for this silver carp.

**Fig 3:**
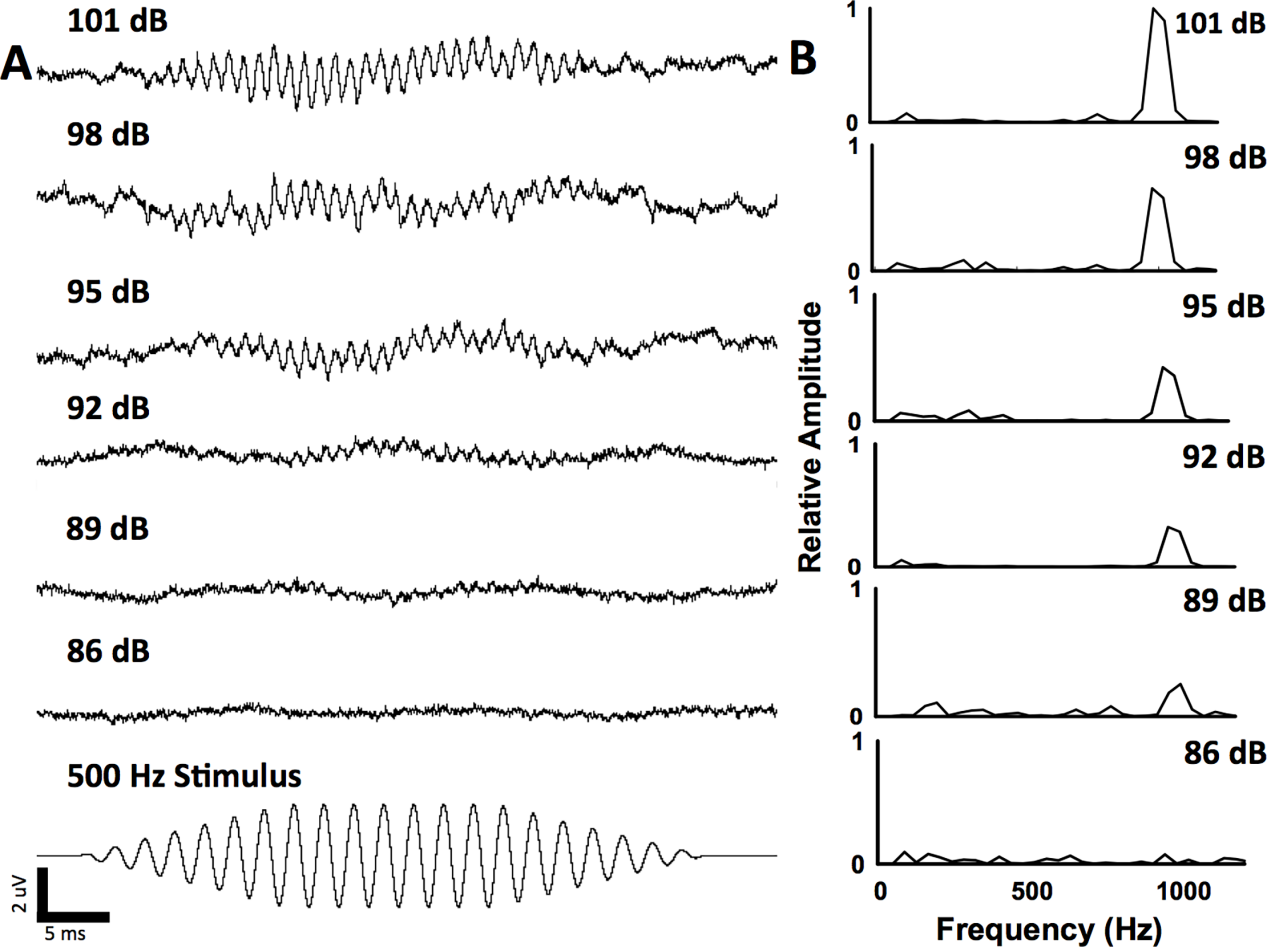
Example auditory evoked potentials (AEP) recorded from a bighead carp at 500 Hz. Averaged AEP traces **(A)** and FFT analysis **(B)** at six different sound pressure levels, including below the hearing threshold (86 dB re 1 μPa SPL_rms_). FFT peaks are two times the stimulus frequency (1000 Hz). Hearing threshold was 89 dB re 1 μPa SPL_rms_ for this bighead carp.

The ambient sound pressure level was 70 dB re 1 μPa SPL_rms_ for all experiments and the baseline particle acceleration level was -96.0 dB re 1 ms^-2^. The acoustic impedance at all three sound pressure levels examined indicates that there were no major resonances in the tank at the test frequencies (Fig 1A). At 130 dB re 1 μPa SPL_rms_, the dorsoventral (z: -51.6 ± 7.0 dB re 1 ms^-2^) axis had the highest mean particle acceleration across all test frequencies compared with the x (-54.0 ± 9.0 dB re 1 ms^-2^) and y (-58.7 ± 6.2 dB re 1 ms^-2^) axes. Fig 1B shows the individual the acceleration levels in the x, y, and z-axes for all of the test frequencies at 130 dB re 1 μPa SPL_rms_. Auditory evoked potentials were recorded for all carp species from 100 Hz to 5 kHz and the waveforms were similar across species type at each frequency. Figs 2 and 3 show representative AEP traces from a silver and bighead carp, respectfully.

**Fig 4:**
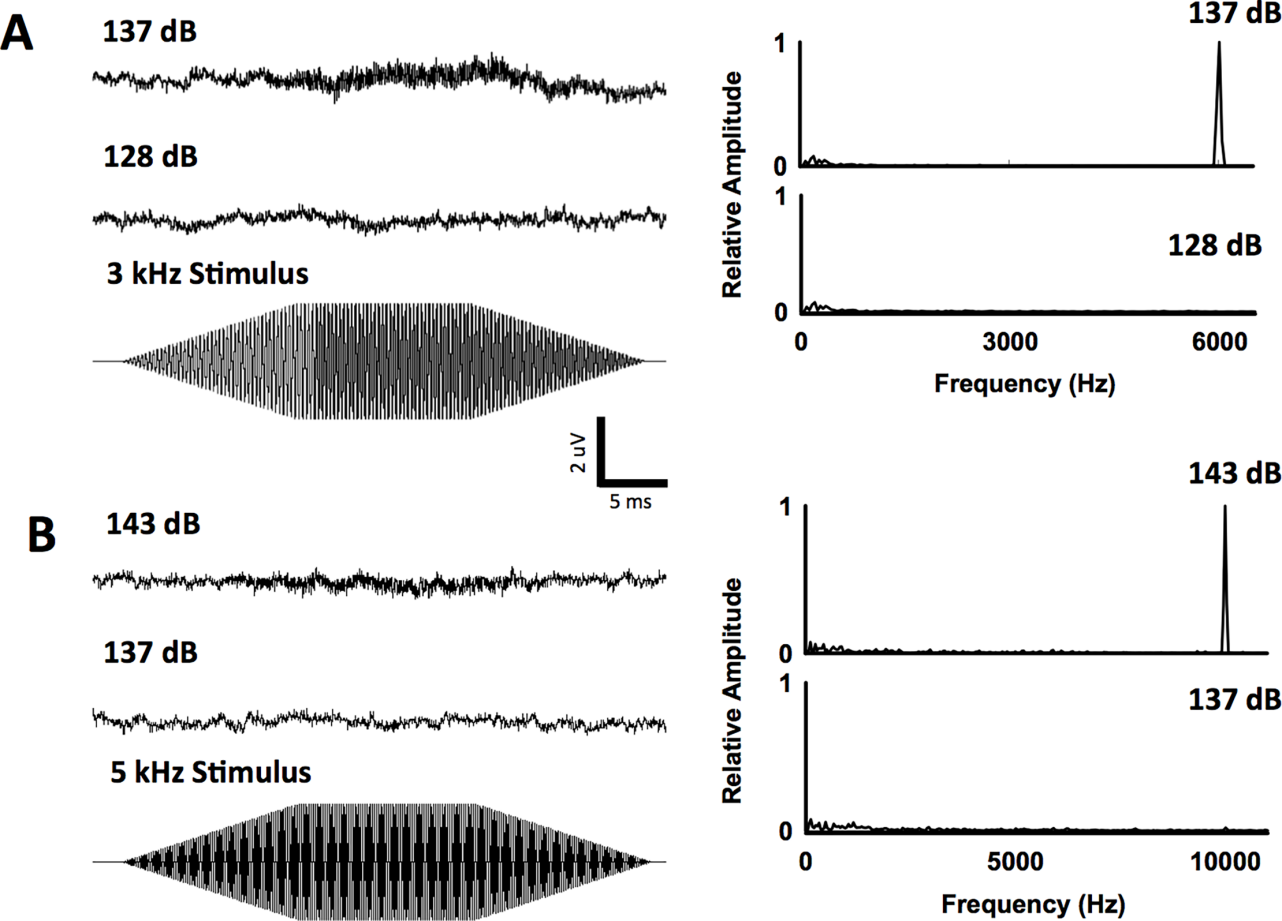
Example AEP traces recorded in response to high frequencies. Examples of AEPs (with FFT analysis) elicited at 3 kHz from a silver carp **(A)** and 5 kHz from a bighead carp **(B)**; upper traces were taken at sound pressure levels above the hearing threshold while the lower traces represent a baseline recorded at sound pressure levels below the hearing threshold.

**Fig 5:**
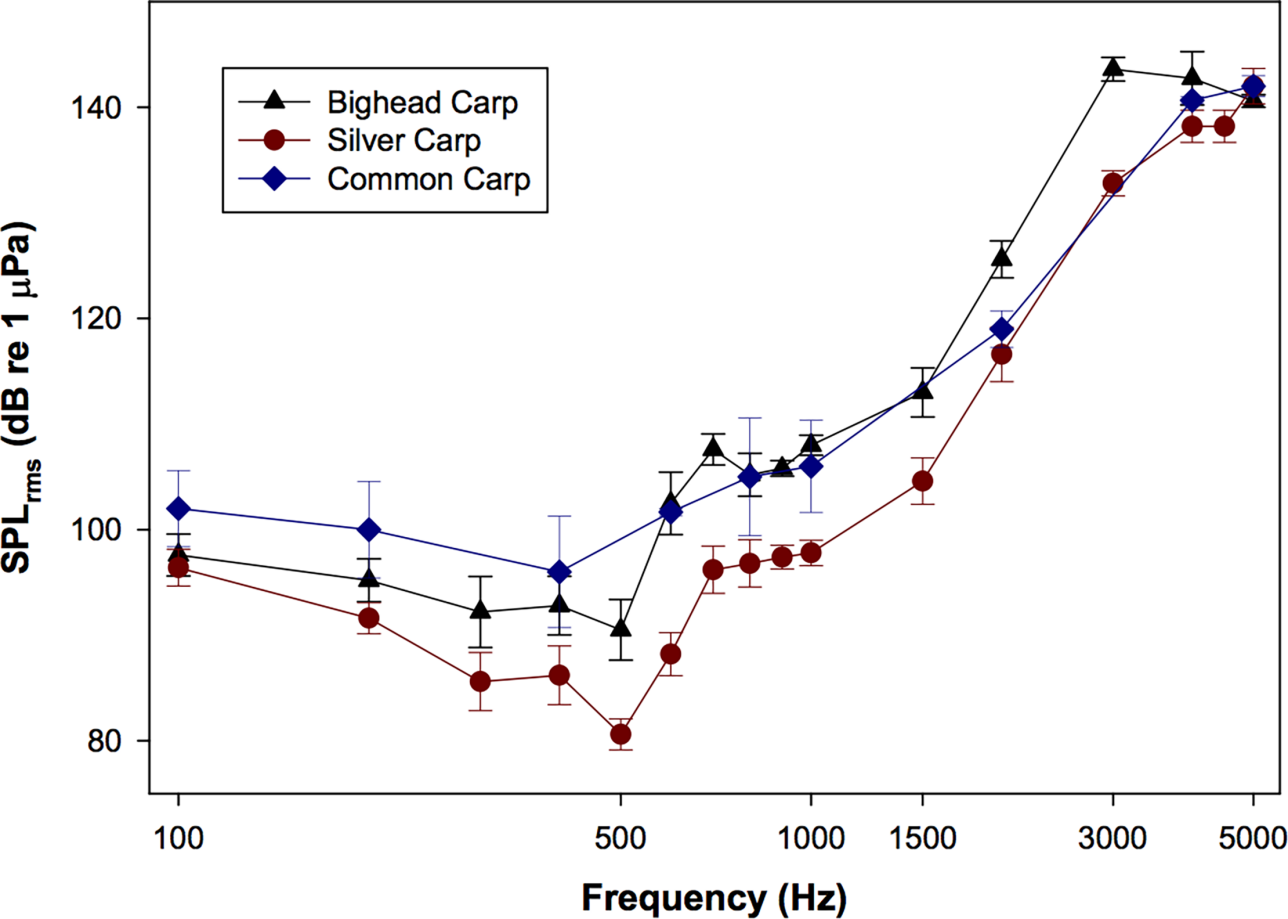
Audiogram for bighead, silver, and common carp. Each data point represents the minimum sound pressure level (SPL; dB re 1 μPa SPL_rms_) necessary to invoke an AEP response at each frequency examined (100 Hz– 5 kHz). Data are plotted as mean ± SD. Silver carp had the lowest thresholds of the species examined.

For silver carp, AEPs were recorded up to 5 kHz (threshold: 142 ± 3.7 dB re 1 μPa SPL_rms_; Table 1, Figs 4A and 5). The lowest mean threshold for silver carp was at 500 Hz (threshold: 80.6 ± 3.3 dB re 1 μPa SPL_rms_; Table 1, Figs 2 and 5). Similarly, AEPs were recorded for bighead carp up to 5 kHz (threshold: 140.6 ± 1.3 dB re 1 μPa SPL_rms_; Table 1, Figs 4B and 5) with the lowest mean threshold at 500 Hz (threshold: 90.5 ± 5.8 dB re 1 μPa SPL_rms_; Table 1, Figs 3 and 5). For common carp, the lowest mean threshold was at 400 Hz (threshold: 96.0 ± 9.2 dB re 1 μPa SPL_rms_; Table 1, Fig 5). Silver carp had significantly lower (F_4,38_ = 70.46; P < 0.05) thresholds than bighead and common carp at 500, 600, 700, 900, 1000, 1500, and 3000 Hz (Table 1). At 2000 Hz, the mean threshold for silver carp was significantly lower (P < 0.05) than that of bighead carp but not common carp (Table 1). Common carp and bighead carp were also not significantly different at 2000 Hz.

**Table 1:** Mean sound pressure and particle motion thresholds for each species at all frequencies examined. Letters indicate significant groups and * indicates significantly lower mean thresholds (ANOVA P < 0.05). BHC = bighead carp; SVC = silver carp; CC = common carp.

Species/Frequency (Hz)	Mean Threshold SPL (dB re 1 μPa) ± 1 SD	Mean Threshold Particle Motion (dB re 1 ms^-2^) ± 1 SD
BHC 100	97.6 ± 4.5	-64.2 ± 3.5
SVC 100	96.4 ± 3.9	-65.2 ± 3.1
CC 100	102.0 ± 6.3	-60.7 ± 5.0
BHC 200	95.2 ± 4.6	-66.6 ± 3.1
SVC 200	91.6 ± 3.3	-69.1 ± 2.2
CC 200	100.0 ± 8.0	-63.4 ± 5.4
BHC 300	92.2 ± 7.5	-63.6 ± 6.0
SVC 300	85.6 ± 6.2	-68.8 ± 4.9
BHC 400	92.8 ±6.2	-73.2 ± 3.6
SVC 400	86.2 ± 6.2	-77.0 ± 3.6
CC 400	96.0 ± 9.2	-71.3 ± 5.3
BHC 500	90.5 ± 5.8	-67.2 ± 3.1
SVC 500	80.6 ± 3.3*	-72.5 ± 1.8*
BHC 600	102.5 ± 5.9	-61.8 ± 2.6
SVC 600	88.2 ± 4.6*	-68.0 ± 1.9*
CC 600	101.7 ± 0.58	-62.2 ± 0.25
BHC 700	107.6 ± 3.3	-67.3 ± 2.2
SVC 700	96.2 ± 5.0*	-75.0 ± 3.4*
BHC 800	105.2 ± 4.6	-65.7 ± 3.2
SVC 800	96.8 ± 5.0	-71.6 ± 3.5
CC 800	105.0 ± 9.6	-65.8 ± 6.8
BHC 900	105.8 ± 1.6	-59.7 ± 1.1
SVC 900	97.4 ± 2.5*	-65.3 ± 1.7*
BHC 1000	108.0 ± 2.1	-66.6 ± 1.8
SVC 1000	97.8 ± 2.7*	-75.1 ± 2.2*
CC 1000	106.0 ± 1.6	-68.2 ± 6.3
BHC 1500	113.0 ± 5.2	-54.6 ± 2.5
SVC 1500	104.6 ± 4.9*	-58.7 ± 2.4*
BHC 2000	125.6 ± 3.9^a^	-57.0 ± 2.3^a^
SVC 2000	116.6 ± 5.8^b^	-62.3 ± 3.4^b^
CC 2000	119.0 ± 3.0^ab^	-60.87 ± 1.8^ab^
BHC 3000	143.6 ± 2.5	-42.6 ± 1.7
SVC 3000	132.8 ± 2.7*	-50.0 ± 1.8*
BHC 4000	142.8 ± 5.1	-49.7 ± 2.4
SVC 4000	138.2 ± 3.4	-51.9 ± 1.6
CC 4000	140.7 ± 0.58	-50.7 ± 0.27
BHC 5000	140.6 ± 1.3	-50.8 ± 0.45
SVC 5000	142.0 ± 3.7	-48.6 ± 4.9
CC 5000	142.0 ± 1.7	-50.3 ± 0.58

**Fig 6:**
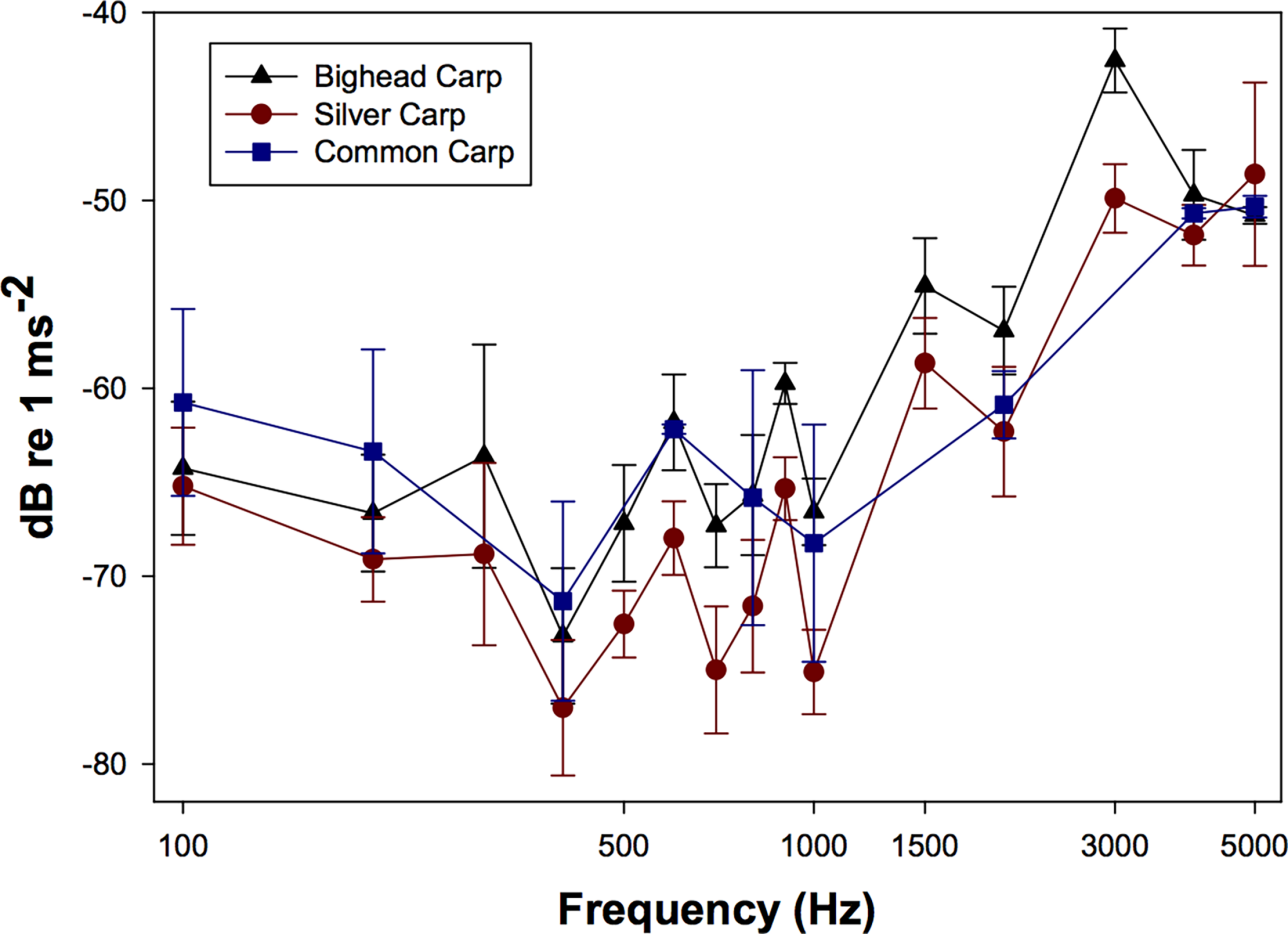
Particle acceleration thresholds (dB re 1 ms^-2^) for the bighead, silver, and common carp. Each threshold was derived using a tri-axial accelerometer and are reported as the combined magnitude vector of the x, y, and z-axes Data are reported as mean (± SD).

Acceleration thresholds were lowest between 400–1000 Hz for all three species (Fig 6). Silver carp had significantly lower (F_4,38_ = 32.26, P < 0.05) mean acceleration thresholds at 500, 600, 700, 900, 1000, 1500, and 3000 Hz (Table 1, Fig 6) than bighead or common carp. Similar to the sound pressure thresholds, common carp thresholds were not significantly different from either silver or bighead carp thresholds at 2000 Hz, but silver carp had significantly lower thresholds than bighead carp at this frequency (Table 1, Fig 6). The lowest mean acceleration threshold for all three species was at 400 Hz (silver carp: -77.0 ± 3.6 dB re 1 ms^-2^; bighead carp: -73.2 ± 3.6 dB re 1 ms^-2^; common carp: -71.3 ± 5.3 dB re 1 ms^-2^; Table 1, Fig 6).

### Discussion

Auditory evoked potentials were elicited from silver and bighead carp between 100 Hz– 5 kHz. The lowest mean sound pressure threshold was 500 Hz for both species. This demonstrates that bigheaded carp can detect higher frequencies than originally reported, which will be important in the future design of acoustic deterrents. Finally, the acoustic impedance of the tank was also determined and is reported so that it can be used in comparison with future fish hearing studies.

The need for investigating the upper range of bigheaded carp hearing originates from behavioral studies examining silver and bighead carp responses to pure tones (500–2000 Hz) and broadband sound (0.06–10 kHz) recorded from an outboard motor. Both species showed negative phonotaxis to pure tone stimuli (150 dB re 1 μPa SPL_rms_) in outdoor concrete ponds but habituated to these pure tone sounds after a few presentations [14, 15]. However, they demonstrated consistent negative phonotaxis to the broadband sound stimulus. Furthermore, the broadband sound also deterred both species from crossing a narrow opening in a concrete barrier [10]. While these studies demonstrate that the outboard motor recording is effective in altering bigheaded carp swimming, it is unclear what frequency components contained in the broadband sound impact fish behavior. The study by Lovell et al. [16] first characterized bigheaded carp hearing ability, but the researchers did not report thresholds greater than 3 kHz. Therefore the upper range of bigheaded carp hearing needed to be determined and was hypothesized to include higher frequencies than previously reported because (1) AEPs have been elicited at 5 kHz in other cyprinids [17] and (2) the behavior experiments demonstrated negative phonotaxis to broadband outboard motor recordings which had energy in frequencies up to 10 kHz. The present results indicate that both silver and bighead carp can detect a broad range of frequencies from 100 Hz up to 5 kHz with lowest thresholds at 500 Hz.

The tuning curves from the present study and those reported by Lovell et al. [16] demonstrate a broad sensitivity with the lowest thresholds between 300 Hz and 1.5 kHz for both bigheaded carp species. However, Lovell et al. [16] found higher hearing thresholds and different peak sensitivities (bighead carp: 1500 Hz; silver carp: 750 Hz). Comparisons between AEP results are challenging because there are many variables between experimental design and methodology [21]. The present study used a single speaker that could produce greater sound pressure levels (153 dB re 1 μPa at 150 Hz) compared to the twin speakers used by Lovell et al. [16], which did not have an output greater than 134 dB re 1 μPa. Additionally, in the present study, both the visual and FFT analysis were used to determine the threshold sound pressure level for each frequency while Lovell et al. [16] only employed visually determined thresholds, which can be more subjective [21].

While comparisons between AEP thresholds generated using different setups can be difficult [21], several common carp were tested to qualitatively determine similarities in tuning curve shape and peak frequency detection between the current study and the published literature. The present findings show similar peak thresholds (400 Hz; [23]) and tuning curve shape as other AEP studies conducted on common carp [25, 23]. This suggests reliability of the AEP method utilized in the present study.

In addition to reporting AEP-derived threshold curves for the three carp species in regards to sound pressure, the tuning curves for acoustic particle motion were also determined. For all three species, the lowest mean particle acceleration thresholds were at 400 Hz. It is believed that all fish are capable of detecting acoustic particle motion through direct stimulation of the otoliths [33, 34]. However, many AEP studies only report sound pressure thresholds. As carp are likely capable of detecting both particle motion and sound pressure, the threshold curves for both were characterized and reported.

**Fig 7:**
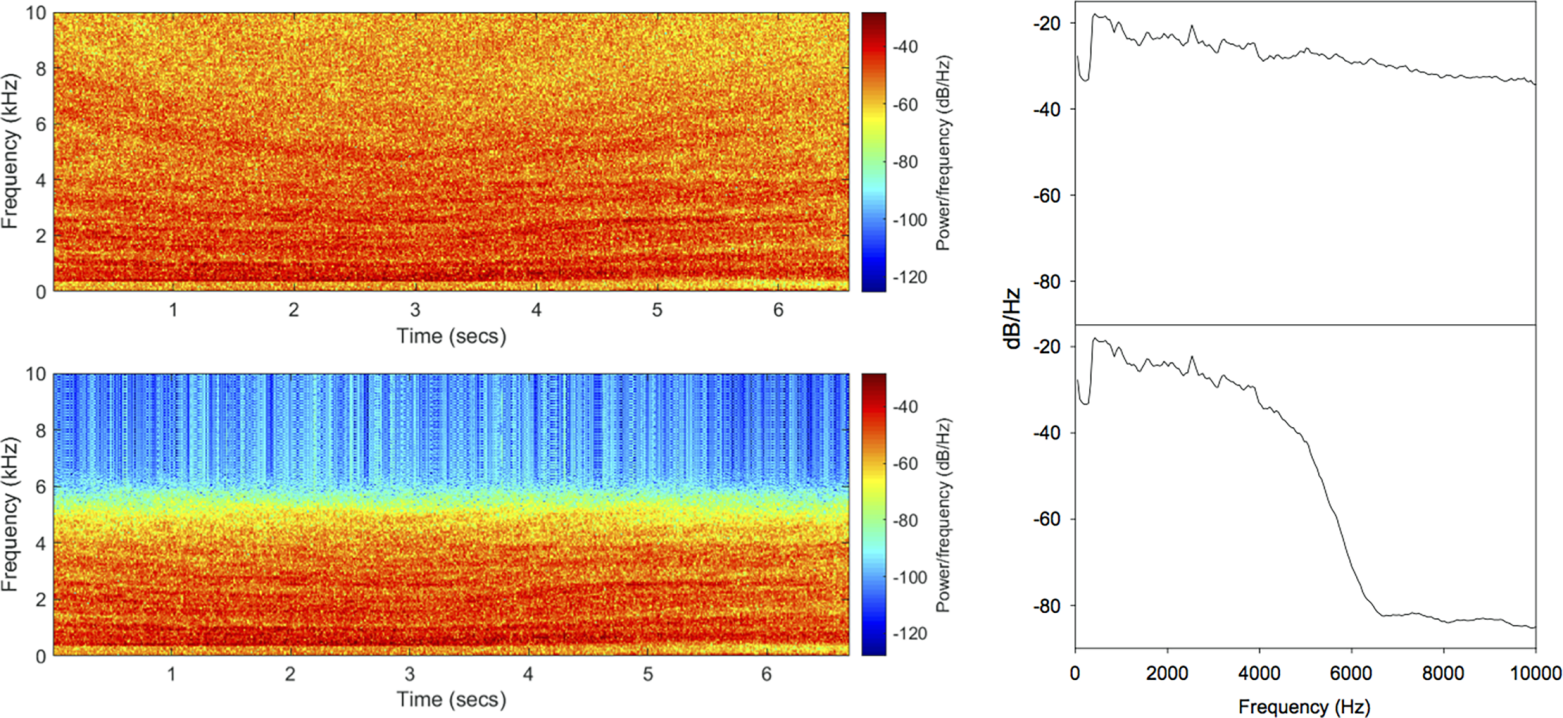
Low-pass filtered broadband sound. Spectrogram (left) and power spectrum (right) of the unfiltered (top) broadband sound used by Vetter et al. [14, 15] and Murchy et al. [10] to deter bigheaded carp. Bottom spectrogram and power spectrum represent the same broadband sound with a 5 kHz low-pass filter applied using Audacity (version 2). Spectrograms were generated using MatLab (version 9.3) and power spectra were analyzed in Audacity.

Recent reviews comparing the AEP and behavioral paradigms have concluded that while the two methods yield similar frequency ranges, the thresholds vary greatly, even among AEP studies [17, 21]. The AEP technique is therefore most useful as a means to determine range of frequencies that can be detected by a fish species’ auditory end organs. In the Vetter et al. [14, 15] studies, although the fish demonstrated a robust avoidance response, the stimulus was a broadband sound and therefore the results could not identify a specific frequency or range of frequencies that most affected fish behavior. Ideally, to evaluate effective deterrent sound stimuli, these behavioral experiments would be repeated with many more frequencies examined. However, this would be logistically difficult as these studies were conducted in large outdoor ponds that take multiple days to fill and drain and require a lengthy acclimation period for the fish. Furthermore, there are an infinite number of sound combinations that could be examined. Based on the results of the present study, these assessments can focus on frequencies between 100 Hz to 5 kHz and it is now imperative that behavioral studies examining bigheaded carp responses to sound be evaluated to better understand bigheaded carp hearing and to develop the most effective acoustic deterrent. In addition to examining the response behavior in fish exposed to a range of high frequency pure tones, applying a 5 kHz low-pass filter to the broadband sound will allow more energy to be broadcast in the hearing range of the bigheaded carp and may provide greater deterrence (Fig 7).

Finally, it is possible that bigheaded carp can hear frequencies above 5 kHz at sound pressure levels > 150 dB re 1 μPa SPL_rms_, as other AEP studies have shown that ostariophysans can detect up to ~8 kHz [17]. When exposed to high sound pressure levels (i.e. 140–149 dB re 1 μPa SPL_rms_) at frequencies between 6–9 kHz, small peaks on the FFT at two times the stimulus frequency were observed for some fish at 6, 7, 8, and 9 kHz, but these peaks appeared inconsistently and did not meet the established AEP criterion of the present study. However, given the constraints of tank size and speaker output, generating sound pressure levels above 150 dB re 1 μPa SPL_rms_ were not possible. Additional research could examine sensitivity to frequencies > 5 kHz at sound pressure levels above 150 dB re 1 μPa SPL_rms_ in both carp species.

The results of the present study provide important insight on the upper range of silver and bighead carp hearing, as they indicate higher frequency hearing than has been previously reported. Together with findings that bigheaded carp behavior can be modified using broadband sound, this research will aid in developing an effective acoustic deterrent. Particularly, the conclusion that bigheaded carp hearing extends up to 5 kHz could allow for refinement of the broadband stimulus to target bigheaded carp. Further research may allow for development of an effective acoustic deterrent primarily comprised of frequencies above the hearing range of non-ostariophysans, however care must to be taken to avoid disturbing native cyprinids.
